# Practical inference for a complier average causal effect in cluster randomised trials with a binary outcome

**DOI:** 10.1177/17407745251378407

**Published:** 2025-10-16

**Authors:** Tansy Edwards, Jennifer Thompson, Charles Opondo, Elizabeth Allen

**Affiliations:** 1 MRC International Statistics and Epidemiology Group, Faculty of Epidemiology and Population Health, London School of Hygiene and Tropical Medicine, London, UK; 2 Department of Medical Statistics, Faculty of Epidemiology and Population Health, London School of Hygiene and Tropical Medicine, London, UK

**Keywords:** complier average causal effect, cluster randomised trial, compliance, adherence

## Abstract

**Background:**

Individual non-compliance with an intervention in cluster randomised trials can occur and estimating an intervention effect according to intention-to-treat ignores non-compliance and underestimates efficacy. The effect of the intervention among compliers (the complier average causal effect) provides an unbiased estimate of efficacy but inference can be complex in cluster randomised trials.

**Methods:**

We evaluated the performance of a pragmatic bootstrapping approach accounting for clustering to obtain a 95% confidence interval (CI) for a CACE for cluster randomised trials with monotonicity and one-sided non-compliance. We investigated a variety of scenarios for correlated cluster-level prevalence of a binary outcome and non-compliance (5%, 10%, 20%, 30%, 40%). Cluster randomised trials were simulated with the minimum number of clusters to provide at least 80% and at least 90% power, to detect an ITT odds ratio (OR) of 0.5 with 100 individuals per cluster.

**Results:**

Under all non-compliance scenarios (5%–40%), there was negligible bias for the CACE. In the worst-case of bias, a true OR of 0.18 was estimated as 0.15 for the rarest outcome (5%) and highest non-compliance (40%). There was no under-coverage of bootstrap CIs. CIs were the correct width for an outcome prevalence of 20%–40% but too wide for a less common outcome. Loss of power for a CACE bootstrap analysis versus ITT regression analysis increased as the prevalence of the outcome decreased across all non-compliance scenarios, particularly for an outcome prevalence of less than 20%.

**Conclusions:**

Our bootstrapping approach provides an accessible and computationally simple method to evaluate efficacy in support of ITT analyses in cluster randomised trials.

## Background/aim

Cluster randomised trials (CRTs) are often used to evaluate the impact of an intervention that is made available to all eligible individuals in a cluster, for example a community or school. Intention-to-treat (ITT) analyses estimate the effectiveness of the intervention,^1^ which could be combination of the direct of the intervention in those who receive it, other possible indirect effects and the success of the delivery of the intervention to the target population.

There is increasing interest in estimating the efficacy of the intervention in CRTs, that is, the direct effect in individuals who actually receive the intervention. This is to better understand how an intervention is having an impact in individuals and support evidence obtained from ITT analyses.

A method to calculate a complier average causal effect (CACE) point estimate, as an *unbiased* estimate of efficacy in individually and cluster randomised trials has been proposed repeatedly in the literature,^2–5^ under a variety of assumptions that utilise the properties of the randomisation. This method was developed under the principal stratification approach, which can also be used to address challenges of non-compliance and also survivor average causal effects when truncation by death occurs prior to measurement of the outcome of interest.^6,7^ A standard calculation for a confidence interval around this CACE point estimate in individually randomised trials would not be valid for a CRT, as a CRT analysis must account for clustering in the data. Inference to obtain a 95% confidence interval around a CACE is complex in the presence of clustering and more so for binary outcomes.^5,8–10^ Application of existing methods such as instrumental variables and structural equation model regression approaches could be used to obtain a CACE and corresponding confidence interval to account for clustering, however these methods can be relatively computationally and methodologically challenging.^11,12^

In this article, we use simulations to evaluate the performance, in terms of power, coverage, and bias, of a simple, pragmatic and accessible bootstrapping approach for inference for a CACE in CRTs. To illustrate our proposed approach and investigate performance we consider a CRT design with a binary outcome, where clusters in one arm are allocated to receive an intervention and clusters in the other arm receive no intervention (monotonicity and one-sided non-compliance). We apply our proposed approach to simulated CRTs that have sufficient numbers of clusters to be powered to detect a pre-specified ITT effect odds ratio, under a range of scenarios for prevalence of a binary outcome in the control arm with no intervention and prevalence of non-compliance. For simplicity, our simulation design assumes no association between compliance status and baseline covariates. We investigate how well our proposed accessible approach performs for a binary outcome as prevalence of the outcome becomes increasingly rare, in order to understand when this approach can provide evidence of a direct benefit of an intervention in support of an ITT effect.

## Methods

### Trial design

A parallel two-arm superiority CRT with clusters randomly allocated 1:1 to intervention or control (no intervention).

### Primary trial outcome

A binary indicator of the presence or absence of a negative health outcome of interest, for example, infection status, disease status or another poor vs successful prognosis indicator. We pre-specified an ITT odds ratio of 0.5 for a 50% reduction in the outcome in the intervention arm versus the control arm at follow-up and an equal cluster size of 100 individuals per cluster. Throughout the paper, we use ‘outcome’ to refer to the binary outcome of the trial. An equal cluster size was arbitrarily selected based on published CRTs evaluating mass antibiotic distribution for trachoma.^13,14^

### Non-compliance

If a participant in an intervention cluster eligible to receive the intervention did not receive the intervention offered to their cluster, this is non-compliance. We recognise that other terminology may be used or deemed appropriate dependent on context, e.g. non-adherence or non-participation.^15^

### CACE estimation

The CACE is the population-average effect of the intervention in those who take it, i.e. the effect of the intervention in the compliers.^2^ We provide a general explanation and illustration of how to calculate a CACE as an odds ratio (OR) from observed data in the intervention and control arms of a trial, under an established approach that has been illustrated several times in the literature, and the key underlying assumptions made. More detailed theoretical explanations of the derivation and important assumptions are available elsewhere.^3,5^

As most simply outlined in Sommer & Zeger, the observed data by compliance status in the intervention arm and the overall data in the control arm (compliance not observed) are used, under assumptions that rely on the properties of the randomisation, to calculate the CACE.^2^

A key assumption is that compliance status can be considered a pre-randomisation characteristic of individuals, referred to in the literature as *principal compliance status* under the causal inference of potential outcomes framework.^3,10^ In brief, these possible underlying principal compliance status categories are (as illustrated in Table 1) *‘complier’* (would always comply with the allocation they are assigned to), *‘always-taker’* (would always take the intervention offered or seek to receive it, regardless of trial allocation), ‘*never-taker*’ (would never accept/take the intervention offered regardless of allocation) or *‘defier’* (would always follow the opposite to their allocation).

**Table 1.** *Principal compliance* status categories and subsequent possible observed compliance categories when comparing an intervention arm to a control arm with no intervention.

	Arm	“Users”	“Non-users”
*Principal compliance status*	Control arm: no intervention	*always-takers, defiers*	*compliers, never-takers*
	Intervention arm	*compliers, always-takers*	*never-takers, defiers*
**In a parallel arm trial comparing an intervention arm to a control arm of no intervention or access to intervention:**			
Observed compliance	Control arm: no intervention	Do not receive intervention	Do not receive intervention
	Intervention arm	Compliance	Non-compliance

Using the same terminology as Gruber et al;^4^ Users receive/take the intervention, Non-users do not. Compliers will follow (comply with) their randomised assignment. Always-takers will accept the intervention regardless of randomised assignment. Never-takers will never accept the intervention, regardless of randomised assignment. Defiers do the opposite of their randomised assignment and are assumed not to occur. In a trial where the control arm is no intervention and the control arm cannot access the intervention, the always-taker category does not occur; that is, only *principal compliers* and *never-takers* can occur.

A key assumption when applying this methodology is that there are no defiers^3^ as defiers would be unlikely to consent to participate in a trial. In many trial settings, the control arm consists of no intervention and the control arm participants cannot access the intervention: this excludes a possibility of *always-takers* accessing the intervention. Only *principal compliance* categories of *compliers* and *never-takers* can occur in each arm under our imposed scenarios with monotonicity and one-sided non-compliance. The approach also requires the assumption that there is no treatment effect in never-takers (known as the exclusion restriction).

The outcome data can be tabulated according to observed compliance status and overall in the intervention arm but only overall in the control arm. Table 2 shows hypothetical data from one simulated CRT to illustrate observed and unobserved data and the process for calculation of the ITT OR and CACE. For each observed compliance status category in the intervention arm, the number of individuals at risk of the outcome, the number of individuals with the outcome at follow-up and the risk are tabulated. In the first stage of the calculations (Table 2, cell A), it is assumed that there is a comparable group of non-compliers (‘never-takers’) in the control arm, achieved by the balance of the randomisation. Under this assumption, it follows that the risk (prevalence) of the outcome in the non-compliers is the same in each arm. Also, that the proportion of non-compliers in each arm is the same (Table 2, cell B). This then allows us to calculate, for the control arm, the number of non-compliers at risk and the number of non-compliers with the outcome. These values are then deducted from the total number at-risk and number with the outcome from the totals, to get values for assumed comparable compliers in the intervention arm. It follows that we are able to calculate the CACE in compliers in each arm, from the observed risk of the outcome in the compliers in the intervention arm and the assumed group of comparable compliers in the control arm.

**Table 2.** Example of calculation of the CACE using observed data in an example trial with fixed parameters of 30% prevalence of the outcome in the control arm and 10% non-compliance.

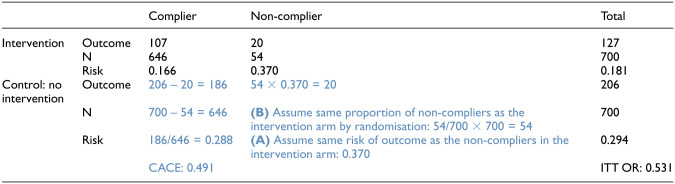				

ITT = intention-to-treat, CACE = complier average causal effect, black: ‘observed’ data, blue: assumed values. OR = odds ratio. Based on 14 clusters (seven per arm) with 100 individuals per arm for 80% power to detect an ITT OR of 0.5.

Note that for a risk difference, this method is equivalent to an established formula for the CACE risk difference; as the ITT risk difference divided by the proportion of compliers.^5^

### A 95% CI around the CACE with clustered bootstraps

Applying standard formulae for calculation of a standard error and confidence interval for an odds ratio would ignore the clustering present in the data, underestimate variance and overestimate significance. So, we used a bootstrapping procedure that allows for clustering during bootstrap sampling to obtain a 95% CI for the CACE.

Random bootstrap samples of clusters were drawn with replacement within each intervention arm. Within clusters, samples of individuals were drawn with replacement within observed compliance strata (intervention arm: complier or non-complier, control arm: overall). The sampling distribution of the CACE, calculated as above, from 1000 replications was used to obtain the 2.5^th^ and 97.5^th^ percentiles as the lower and upper 95% CI bounds, in case of skewness in the sampling distribution. The sampling distribution of the CACE for the trial example in Table 2 is shown in Supplemental Figure S1.

### Simulation study methods

To assess the performance of our analysis approach for inference accounting for clustering, we simulated data from CRTs under a variety of scenarios for both prevalence of the outcome and prevalence of non-compliance, according ADEMP for simulation studies as recommended by Morris et al.^16^

### Data generating mechanism

We began by specifying the cluster-level mean prevalence of the outcome in the control arm, as 5% and the overall cluster-level mean prevalence of non-compliance (all clusters) as 5%.

We generated individual-level compliance and outcome status at follow-up in each arm using Beta-binomial distributions.^17^ The compliance and outcome indicators in the resulting dataset had beta-distributed cluster-level proportions for correlated compliance and outcome, and binomial-distributed individual level disease outcome status within a cluster corresponding to their cluster’s cluster-level proportion of the outcome (supplementary methods). Within clusters, individuals’ compliance and outcome were sample independently without considering any individual level covariates. Individuals in control clusters and non-compliers in intervention arm clusters had their cluster’s untreated probability of the outcome. Individuals in the intervention clusters that were adherent to intervention had their cluster’s treated probability of outcome.

We imposed a between-cluster coefficient of variation (CV) of 0.2 on the prevalence of the outcome without intervention, and set it to be correlated with non-compliance (correlation of 0.5 in bivariate random normal correlated cluster-level data prior to transformation to beta-binomial distribution), so that clusters with a higher prevalence of the outcome had a higher prevalence of non-compliance. For simplicity, our simulation design assumes no association between compliance status and baseline covariates.

For a constant true ITT odds ratio of 0.5, we calculated the *true* CACE odds ratio corresponding to this algebraically (supplementary methods), given the prevalence of outcome and the prevalence of non-compliance.

We used simulations to determine the minimum number of clusters required for at least 80%, and again for least 90%, to detect the ITT effect in the presence of non-compliance. This was to ensure that our simulation study framework was based on investigating the performance of our approach for CRTs with confirmed power to detect an ITT effect in the presence of non-compliance, under the assumptions of an ITT odds ratio of 0.5 and 100 individuals per cluster and each combination of pre-specified values for the prevalence of the outcome and prevalence of non-compliance. The rationale for this was that CRTs without sufficient power for ITT analyses in the presence of non-compliance would not have power for a CACE analysis. The scenarios where trials were designed to have at least 80% power had between 14 and 18 clusters for a prevalence of the outcome in the control arm of 10%–40%. The scenarios where trials were designed to have at least 90% power had between 18 and 24 clusters for a prevalence of the outcome in the control arm of 10%–40% (Supplemental Table S2).

We repeated this process for mean cluster-level prevalence of the outcome of 5%, 10%, 20%, 30% and 40% and applied a prevalence of non-compliance for each outcome prevalence of 5%, 10%, 20%, 30% and 40% (Table 3).

**Table 3.** Summary of simulation parameters.

Parameter	Description
Number of clusters	Minimum number of clusters to provide (1) 80% power and (2) 90%, to detect intention-to-treat (ITT) effect in the presence of non-compliance. Range 14–34.
ITT odds ratio	0.5
Complier average causal effect (CACE)	Calculated algebraically based on ITT odds ratio (OR) and observed non-compliance
Randomisation (clusters)	Intervention, no intervention, 1:1
Cluster size	100 per cluster (equal)
Outcome prevalence in the control arm (cluster mean)	5%, 10%, 20%, 30%, 40%
Prevalence of non-compliance (cluster mean)	5%, 10%, 20%, 30%, 40%
Cluster-level outcome and non-compliance	Correlated beta-distributed proportions, coefficient of variation 0.2
Individual-level outcome and non-compliance	Binomial-distributed corresponding to cluster-level data

For each scenario, 1000 simulated trials were generated.

### Estimands

The primary estimand of interest was the population-average CACE, as an odds ratio. The ITT OR was secondary.

The ITT odds ratio was estimated from the total observed prevalence of the outcome in each arm; ${\hat{d}}_{C}$ in the outcome in the control arm and ${\hat{d}}_{I}$ in the intervention arm:

$${\hat{\mathrm{OR}}}_{\mathrm{ITT}}=\frac{{\hat{d}}_{I}}{1-{\hat{d}}_{I}}\frac{1-{\hat{d}}_{C}}{{\hat{d}}_{C}}$$

The CACE odds ratio is defined as

$$OR_{\mathrm{CACE}}=\frac{d_{\mathrm{IA}}}{1-d_{\mathrm{IA}}}\frac{1-d_{\mathrm{CA}}}{d_{\mathrm{CA}}}$$

We estimate this using the approach of Sommer and Zeger^2^ with observed prevalence of the outcome in the compliers in the intervention arm ( ${\hat{d}}_{\mathrm{IA}}$ ) and estimate the prevalence of the outcome in the assumed compliers in the control arm ( ${\hat{d}}_{\mathrm{CA}}$ ) as

$${\hat{d}}_{\mathrm{CA}}=\frac{{\hat{d}}_{C}-\left({\hat{a}}_{\mathrm{IN}}{\hat{d}}_{\mathrm{IN}}\right)}{\left(1-{\hat{a}}_{\mathrm{IN}}\right)}$$

where ${\hat{a}}_{\mathrm{IN}}$ is the observed prevalence of compliance in the intervention arm, and ${\hat{d}}_{\mathrm{IN}}$ is the observed prevalence of the outcome of non-compliers in the intervention arm.

### Analysis methods

#### Mixed effects model ITT analysis

To check the simulation process, we used mixed effects logistic regression with a random intercept for cluster and CIs were calculated using a t-distribution (degrees of freedom: number clusters minus 2).

#### CACE

We applied the CACE calculation and bootstrap approach described above. Stata code to obtain the CACE and corresponding bootstrapped 95% CI is provided in the supplementary files.

#### ITT analysis with bootstraps

We also produced bootstrap confidence intervals for the ITT OR accounting for clustering in the same way, in order to further understand power, coverage and bias of the bootstrap application.

### Performance measures

For the odds ratios obtained via the bootstrapped approach, we assessed power, confidence interval coverage and effect estimate bias, and confidence interval widths.^16,18^

Power: proportion of simulated trials in which the bootstrap confidence interval did not include an odds ratio of 1 (the null effect).

Confidence interval coverage: proportion of simulated trials in which the bootstrap confidence interval included the true effect. With 1000 simulations in each scenario, the ideal (95% chance of) coverage of the bootstrap confidence intervals would be between 93.6% and 96.4% based on 1000 simulations.^19^ Above 96.4% is indicative of over-coverage and wider confidence intervals, corresponding to lower power and less precision. Below, 93.6% over-estimation of significance.

Bias: the estimated value from the ‘observed’ data for each simulated trial, minus the true value specified for each outcome-prevalence scenario (log scale).

Three confidence interval width ratios were calculated to further understand performance; (a) observed (bootstrapped) ITT to ITT regression, (b) CACE to ITT regression and (c) CACE to observed ITT.

## Results

### Power

**Figure 1.**
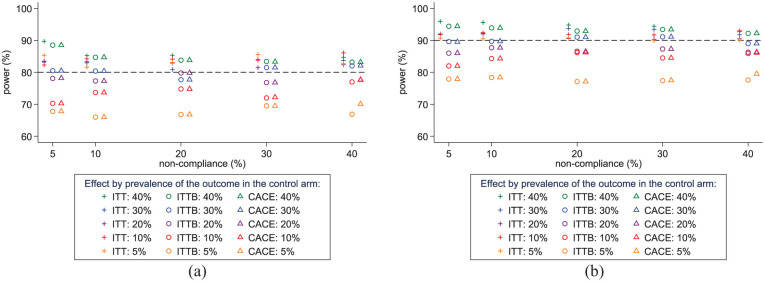
Power of the bootstrapped approach to detect the CACE in CRTs powered to detect an ITT effect in the presence of non-compliance, by prevalence of the outcome in the control arm. (a) CRTs with at least 80% power to detect an ITT OR = 0.5 (b) CRTs with at least 90% power to detect an ITT OR = 0.5 ITT = results from regression analysis with t-distribution adjustment, ITTB = observed (bootstrapped) ITT results, CACE = complier average causal effect (bootstrapped)

Comparing power for ITT regression analyses and CACE bootstrap analyses, we observed that the loss of power for CACE increased as the prevalence of the outcome decreased, across all non-compliance scenarios (Figure 1). This was particularly apparent once the outcome became less common (<20%).

For smaller trials simulated to have 80% power for ITT regression analyses, power to detect the CACE for an outcome prevalence of 30%-40% was between 78% and 89% across all non-compliance scenarios. For rarer outcomes, power to detect a CACE was between 66% and 78% for outcome prevalence of 5%–20% (Figure 1, Supplemental Table 3).

For the larger trials simulated to have 90% power for ITT regression analyses, there was 82%–88% power to detect a CACE for an outcome prevalence of 10%–20%. For a 5% outcome prevalence, power for a CACE was <80% across all non-compliance scenarios (Figure 1, Supplemental Table 4).

The ITT bootstrap analysis had very similar power to the CACE bootstrap analysis in most scenarios This indicates that it is the bootstrap process that leads to the loss of power rather than the reduced sample size for estimation of the CACE.

### Coverage

**Figure 2.**
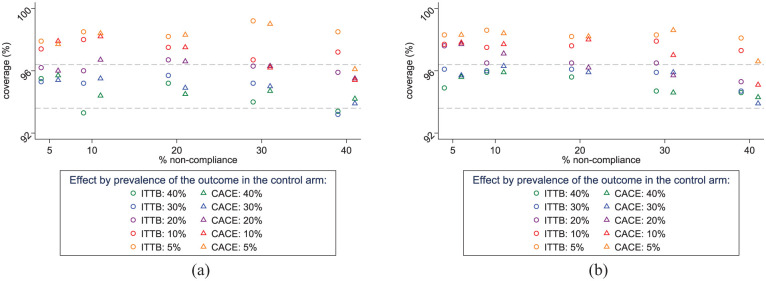
Coverage of the bootstrapped 95% CI for the CACE in CRTs powered to detect an ITT effect in the presence of non-compliance, by prevalence of the outcome in the control arm. (a) CRTs with at least 80% power to detect an ITT OR = 0.5 (b) CRTs with at least 90% power to detect an ITT OR = 0.5. ITTB = observed (bootstrapped) ITT results, CACE = complier average causal effect, CI = confidence interval. Ideal (95% chance of) coverage of the bootstrap confidence intervals is between 93.6% and 96.4%.

CACE bootstrap confidence interval coverage was close to 95%, varying between 94% and 97% for an outcome prevalence of 20% or higher and any level of non-compliance, in the smaller trials with a number of clusters to provide at least 80% power for ITT analyses (Figure 2, Supplemental Table 3).

For lower outcome prevalence (5%–10%), coverage was higher than 95%, varying between 95% and 99%, indicating confidence intervals were too wide. Coverage results were similar for trials designed to have 90% power for ITT regression analyses (Figure 2, Supplemental Table 4).

### Bias

**Figure 3.**
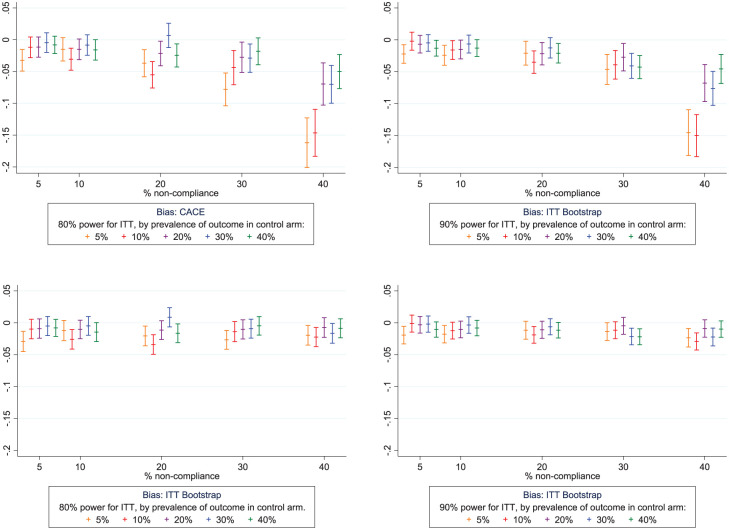
Mean absolute bias and 95% CI (log-odds scale) in bootstrapped results, by power and prevalence of the outcome in the control arm. Legend: ITT = intention-to-treat, CACE = complier average causal effect, CI = confidence interval.

The true CACE is known and algebraically linked to the fixed ITT OR simulation parameter of 0.5 (supplementary methods). There was a small amount of bias away from the null in the estimated CACE intervention effects in the direction of over-estimation of the CACE point estimate (Figure 3, Supplemental Tables 5 and 6). Although increasing patterns of bias were observed for the estimated CACE as non-compliance increased, the bias was still small. For example, the largest bias was seen for the rarest outcome prevalence of 5% and the highest prevalence of non-compliance of 40% in the smaller trials designed to have 80% power (mean estimated CACE = 0.15, true CACE = 0.18, absolute difference on the OR scale of 0.03, mean absolute bias on log scale = −0.145). For the scenarios with the largest bias, there was also over-coverage of the confidence intervals which compensated for the bias.

### Confidence interval width

**Figure 4.**
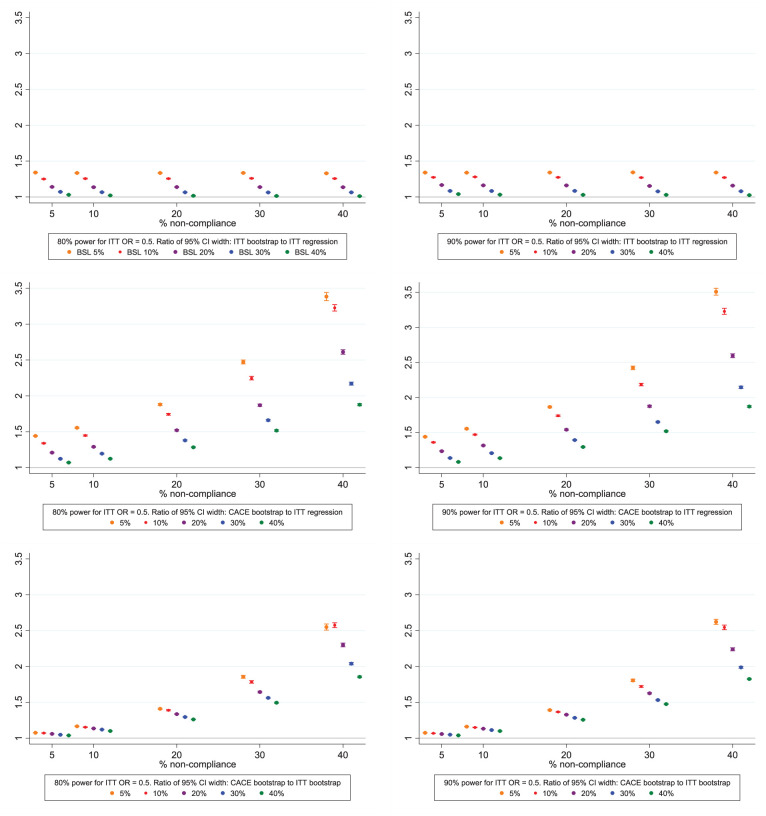
Variability in bootstrapped approach: ratio of CI widths, with 95% CI around the ratio, by power and prevalence of the outcome in the control. Legend: ITT = intention-to-treat, CACE = complier average causal effect, CI = confidence interval.

CACE bootstrap confidence intervals were wider than ITT regression and ITT bootstrap confidence intervals. The difference increased with lower outcome prevalence and higher non-compliance (Figure 4, Supplemental Tables 5 and 6). At the extremes, for smaller trials designed to have 80% power for ITT regression analyses, with 5% outcome prevalence and 40% non-compliance (lowest outcome prevalence, highest non-compliance), confidence intervals were 3.4 times wider for CACE bootstrap and 1.3 times for ITT bootstrap than ITT regression intervals. However, for 40% outcome prevalence and 5% non-compliance (highest outcome prevalence and lowest non-compliance) bootstrap confidence intervals had 1.1 and 1.0 times the width of ITT regression and bootstrap intervals respectively. Results followed a similar pattern for trials designed to have 90% power for ITT regression analyses.

### Application to real data

In a CRT in Malawi, 29 schools were randomised to an intervention to evaluate training teachers to diagnose and treat uncomplicated malaria in their pupils and 29 schools to no intervention.^20^

The primary outcome was absenteeism, captured using daily attendance registers and defined as a proportion (or, percentage) of absences out of the number of eligible days of attendance over term days in a 50 week period (ineligible days during term-time could be term-time public holidays, bad weather school closures, absence for other reasons unrelated to ill-health).

Of interest was the direct intervention effect among pupils who potentially had malaria. The children who attended at least one consultation were the compliers.^21^

The CACE was defined as the odds ratio of absenteeism among compliers in the intervention group relative to an assumed group of compliers in the control arm.

There were 4587 children in the intervention arm and 4430 children in the control arm with daily attendance data available. A total of 47,469 absences were observed over 266,459 follow-up person-days in the intervention arm (17.8%), and 51,503 absences over 256,502 follow-up person-days in the control arm (20.1%).

In the intervention arm, 1945 (42.4%) children were compliers (attended at least on consultation). Absenteeism was 15.6% in the compliers in the intervention arm (18,564 absences out of 118,694 person-days of follow-up). In the non-compliers in the intervention arm, absenteeism was 9.6% (28,905 absences out of 147,765 person-days of follow-up among the 2642 non-compliers). The ITT odds ratio was 0.86, a 14% reduction in odds of absenteeism in the intervention schools versus control schools (95% CI 0.74 to 1.00). The CACE was further from the null (OR = 0.70, 95% CI 0.43 to 1.01, using the clustered bootstrap approach), which alongside the simulation and ITT results, strengthens the findings of the trial and suggests the intervention can have a direct effect in reducing absenteeism in the schools enrolled in the CRT in Malawi.

Applied to this dataset, the bootstrap ITT OR was the same as the maximum likelihood OR from a random effects logistic regression with an almost identical 95% CI (OR = 0.86, 95% CI: 0.73–1.00).

## Conclusion

The performance of a bootstrap approach to obtain a 95% CI around a CACE that accounts for clustering in an analysis of a CRT was explored across scenarios of low- medium prevalence of both the outcome and non-compliance. This was to understand if the approach could be a useful complimentary analysis to ITT and when it may be possible to detect a CACE in support of ITT findings, demonstrating direct benefit of an intervention, especially as the outcome becomes less common. We followed an established approach to calculate a CACE.^2,3,4^ We found that our bootstrapping approach to inference had minimal bias and produced conservative confidence intervals that accounted for clustering in the data across all our scenarios including with a small number of clusters.

For CRTs that had close to 80% power to detect an ITT effect, it was only possible to detect the CACE for more common outcomes (at least 30% outcome prevalence in the control arm) since power to detect the CACE remained close to 80%. To detect a CACE for less common outcome using this method, a CRT will need to have higher power for ITT analyses in the presence of non-compliance. While the confidence intervals for the CACE were always wider than for the ITT analyses, which supports previous literature^3^ that the variance of CACE effects in individually randomised trials is always larger than ITT effects, we found that this was counteracted by the CACE being a larger intervention effect.

The lower power of the CACE bootstrap analysis was largely driven by the percentile bootstrap method, as power was similar between the ITT bootstraps and CACE bootstraps. This may be due to the high coverage of the bootstrap confidence intervals, which has been observed previously for this clustered bootstrap approach in other contexts.^22^ Other bootstrap methods or permutation tests may reduce this loss of power and provide better performance for rare outcomes.^23,24^

The bias in CACE analyses was small and likely due a known small sample bias in log-odds ratios; in a small sample and with low prevalence, the log-odds ratio has not yet converged to a normal distribution, and this results in a bias.^25^

We believe this approach could be broadly applicable to CRTs across a range of settings. In health research, some examples could be mass drug administration of therapeutic treatment intervention to communities or schools to reduce the prevalence of infectious diseases, or child mortality in the case of O’Brien et al,^26^ or vaccination studies that utilise a cluster randomised design.^27^ Outside this field, our approach could be used in education or economics where cluster randomised trials are also commonly used. A limitation of this approach for inference, compared to more complex approaches to inference, is that it does not lend itself easily to adjustment for baseline characteristics, which may be of interest to some researchers. Further research could include evaluating performance of the bootstrap approach with CACE estimators that include other compliance categories, for example if individuals in the control arm had been able to access the intervention, they could be considered as ‘always-takers’, and to compare the performance of our approach to other more complex published methods for cluster randomised trials^28^ to understand the relative benefits of our simpler approach. Extensions to the simulation studies could compare this simple approach to obtaining a CACE, in support of a direct benefit of the intervention in support of ITT results, to more complex structural equation modelling and parametric estimation approaches, that may or may not also account for baseline covariate adjustment,^29^ informative cluster size,^30^ and small cluster sizes. Results of such studies would shed light on whether in the case of rare outcomes, where the bootstrapping approach for the CACE is underpowered, more state-of-the-art complex modelling frameworks can improve precision sufficiently to detect evidence of a CACE.

Using our proposed bootstrapping approach to obtain a confidence interval for a CACE, accounting for clustering, is an accessible and simple method to estimate efficacy in support of effectiveness analyses in CRTs.

## Supplemental Material

sj-docx-1-ctj-10.1177_17407745251378407 – Supplemental material for Practical inference for a complier average causal effect in cluster randomised trials with a binary outcome Supplemental material, sj-docx-1-ctj-10.1177_17407745251378407 for Practical inference for a complier average causal effect in cluster randomised trials with a binary outcome by Tansy Edwards, Jennifer Thompson, Charles Opondo and Elizabeth Allen in Clinical Trials
