# Supplementary material for: Practical inference for a complier average causal effect in cluster randomised trials with a binary outcome
Source: Clin Trials. 2025 Oct 16;23(1):33–42. doi: 10.1177/17407745251378407 (PMC12909608; doi:10.1177/17407745251378407)
Supplement: sj-docx-1-ctj-10.1177_17407745251378407 – Supplemental material for Practical inference for a complier average causal effect in cluster randomised trials with a binary outcome [file sj-docx-1-ctj-10.1177_17407745251378407.docx]

**Supplementary Tables and Figures**

**Supplementary Figure S1. Sampling distribution of the bootstrapped CACE, accounting for clustering**

Legend: Example simulated trial (Table 2) with 30% prevalence of the outcome in the control arm, 10% non-compliance, 14 clusters, 100 individuals per cluster and 80% power. The 95% CI around the CACE of 0.49 is 0.30 to 0.75, based on the 2.5^th^ and 97.5^th^ percentiles of the sampling distribution.

**Table S2. Minimum number of clusters derived by simulation, to provide at least 80% and at least 90% power to detect an odds ratio of 0.5 in an intention-to-treat analysis, with 100 individuals per cluster.**

| Mean cluster level prevalence of infection in the control arm (%) |  | Mean cluster level prevalence of non-compliance | | | | |
| --- | --- | --- | --- | --- | --- | --- |
|  |  | 5 | 10 | 20 | 30 | 40 |
| 40 | ITT OR | 0.5 | 0.5 | 0.5 | 0.5 | 0.5 |
|  | CACE OR | 0.48 | 0.46 | 0.40 | 0.34 | 0.26 |
|  | #(clusters) 80% power | 14 | 14 | 14 | 14 | 14 |
|  | #(clusters) 90% power | 22 | 22 | 22 | 22 | 22 |
| 30 | ITT OR | 0.5 | 0.5 | 0.5 | 0.5 | 0.5 |
|  | CACE OR | 0.48 | 0.45 | 0.40 | 0.33 | 0.24 |
|  | #(clusters) 80% power | 14 | 14 | 14 | 14 | 14 |
|  | #(clusters) 90% power | 18 | 18 | 18 | 18 | 18 |
| 20 | ITT OR | 0.5 | 0.5 | 0.5 | 0.5 | 0.5 |
|  | CACE OR | 0.48 | 0.45 | 0.39 | 0.32 | 0.22 |
|  | #(clusters) 80% power | 14 | 14 | 14 | 14 | 14 |
|  | #(clusters) 90% power | 18 | 18 | 18 | 18 | 18 |
| 10 | ITT OR | 0.5 | 0.5 | 0.5 | 0.5 | 0.5 |
|  | CACE OR | 0.48 | 0.45 | 0.38 | 0.30 | 0.19 |
|  | #(clusters) 80% power | 16 | 16 | 16 | 16 | 16 |
|  | #(clusters) 90% power | 24 | 24 | 24 | 24 | 24 |
| 5 | ITT OR | 0.5 | 0.5 | 0.5 | 0.5 | 0.5 |
|  | CACE OR | 0.47 | 0.45 | 0.38 | 0.29 | 0.18 |
|  | #(clusters) 80% power | 26 | 26 | 26 | 26 | 26 |
|  | #(clusters) 90% power | 34 | 34 | 34 | 34 | 34 |

**Table S3. Mean odds ratios from regression and bootstrap samples, power and coverage from 1000 simulated trials, by prevalence of non-compliance and outcome in the control arm, with at least 80% power to detect an ITT effectiveness OR=0.5**

|  |  | Intention-to-treat OR = 0.5 | | | | Complier average causal effect | | | | |
| --- | --- | --- | --- | --- | --- | --- | --- | --- | --- | --- |
| Control arm prevalence | Non-compliance | Regression OR | Bootstrap OR | Coverage | Bootstrap power | True | Regression OR | Bootstrap OR | Coverage | Bootstrap power |
| 5 | 5 | 0.49 | 0.49 | 97.9 | 67.8 | 0.47 | 0.46 | 0.46 | 97.7 | 67.8 |
| 5 | 10 | 0.50 | 0.49 | 98.5 | 66.0 | 0.45 | 0.44 | 0.44 | 98.4 | 66.0 |
| 5 | 20 | 0.49 | 0.49 | 98.2 | 66.8 | 0.38 | 0.37 | 0.37 | 98.3 | 66.8 |
| 5 | 30 | 0.49 | 0.49 | 99.2 | 69.5 | 0.29 | 0.27 | 0.27 | 99.0 | 69.5 |
| 5 | 40 | 0.49 | 0.49 | 98.5 | 66.9 | 0.18 | 0.16 | 0.15 | 96.1 | 70.1 |
| 10 | 5 | 0.49 | 0.50 | 97.4 | 70.3 | 0.48 | 0.47 | 0.47 | 97.9 | 70.3 |
| 10 | 10 | 0.49 | 0.49 | 98.0 | 73.7 | 0.45 | 0.43 | 0.43 | 98.2 | 73.7 |
| 10 | 20 | 0.48 | 0.48 | 97.5 | 74.8 | 0.38 | 0.36 | 0.36 | 97.5 | 74.8 |
| 10 | 30 | 0.49 | 0.49 | 96.7 | 72.0 | 0.30 | 0.29 | 0.29 | 96.2 | 72.2 |
| 10 | 40 | 0.49 | 0.49 | 97.2 | 77.0 | 0.19 | 0.17 | 0.17 | 95.4 | 77.7 |
| 20 | 5 | 0.53 | 0.50 | 96.2 | 78.1 | 0.48 | 0.47 | 0.47 | 96.0 | 78.2 |
| 20 | 10 | 0.53 | 0.49 | 96.0 | 77.3 | 0.45 | 0.44 | 0.44 | 96.7 | 77.3 |
| 20 | 20 | 0.53 | 0.49 | 96.7 | 79.8 | 0.39 | 0.38 | 0.38 | 96.6 | 79.8 |
| 20 | 30 | 0.50 | 0.49 | 96.3 | 76.8 | 0.32 | 0.30 | 0.31 | 96.3 | 76.8 |
| 20 | 40 | 0.49 | 0.50 | 95.9 | 77.0 | 0.22 | 0.20 | 0.20 | 95.5 | 77.6 |
| 30 | 5 | 0.49 | 0.50 | 95.3 | 80.5 | 0.48 | 0.47 | 0.48 | 95.4 | 80.5 |
| 30 | 10 | 0.49 | 0.50 | 95.2 | 80.4 | 0.45 | 0.44 | 0.45 | 95.5 | 80.4 |
| 30 | 20 | 0.49 | 0.50 | 95.7 | 77.7 | 0.40 | 0.39 | 0.40 | 94.9 | 77.7 |
| 30 | 30 | 0.49 | 0.50 | 95.2 | 81.5 | 0.33 | 0.31 | 0.32 | 95.0 | 81.5 |
| 30 | 40 | 0.48 | 0.49 | 93.2 | 82.0 | 0.24 | 0.21 | 0.23 | 93.9 | 82.1 |
| 40 | 5 | 0.48 | 0.50 | 95.5 | 88.5 | 0.48 | 0.46 | 0.48 | 95.7 | 88.5 |
| 40 | 10 | 0.48 | 0.49 | 93.3 | 84.7 | 0.46 | 0.44 | 0.45 | 94.4 | 84.7 |
| 40 | 20 | 0.48 | 0.49 | 95.2 | 83.8 | 0.40 | 0.38 | 0.39 | 94.5 | 83.8 |
| 40 | 30 | 0.48 | 0.50 | 94.0 | 83.4 | 0.34 | 0.32 | 0.34 | 94.7 | 83.3 |
| 40 | 40 | 0.48 | 0.50 | 93.4 | 83.2 | 0.26 | 0.23 | 0.26 | 94.2 | 83.2 |

Data are mean values from 1000 simulated trials for each combination of control arm prevalence of the outcome and non-compliance

**Table S4. Power and coverage from 1000 simulated trials, by prevalence of non-compliance and outcome in the control arm, with at least 90% power to detect an ITT effectiveness OR=0.5**

|  |  | Intention-to-treat | | | | Complier average causal effect | | | | |
| --- | --- | --- | --- | --- | --- | --- | --- | --- | --- | --- |
| Control arm prevalence | Non-compliance | Regression OR | Bootstrap OR | Coverage | Bootstrap power | True | Regression OR | Bootstrap OR | Coverage | Bootstrap power |
| 5 | 5 | 0.49 | 0.49 | 98.3 | 77.9 | 0.47 | 0.46 | 0.46 | 98.3 | 77.9 |
| 5 | 10 | 0.49 | 0.49 | 98.6 | 78.4 | 0.45 | 0.44 | 0.44 | 98.4 | 78.4 |
| 5 | 20 | 0.49 | 0.49 | 98.2 | 77.1 | 0.38 | 0.37 | 0.37 | 98.2 | 77.1 |
| 5 | 30 | 0.49 | 0.49 | 98.3 | 77.4 | 0.29 | 0.28 | 0.28 | 98.6 | 77.5 |
| 5 | 40 | 0.49 | 0.49 | 98.1 | 77.6 | 0.18 | 0.16 | 0.16 | 96.6 | 79.5 |
| 10 | 5 | 0.50 | 0.50 | 97.7 | 82.0 | 0.48 | 0.47 | 0.47 | 97.8 | 82.0 |
| 10 | 10 | 0.49 | 0.49 | 97.5 | 84.3 | 0.45 | 0.44 | 0.44 | 97.7 | 84.3 |
| 10 | 20 | 0.49 | 0.49 | 97.6 | 86.2 | 0.38 | 0.37 | 0.37 | 98.0 | 86.2 |
| 10 | 30 | 0.49 | 0.49 | 97.9 | 84.5 | 0.30 | 0.29 | 0.29 | 97.0 | 84.5 |
| 10 | 40 | 0.48 | 0.49 | 97.3 | 85.9 | 0.19 | 0.17 | 0.17 | 95.1 | 86.1 |
| 20 | 5 | 0.49 | 0.50 | 97.6 | 86.0 | 0.48 | 0.47 | 0.47 | 97.7 | 86.0 |
| 20 | 10 | 0.49 | 0.49 | 96.5 | 87.7 | 0.45 | 0.44 | 0.44 | 97.1 | 87.7 |
| 20 | 20 | 0.49 | 0.49 | 96.5 | 86.6 | 0.39 | 0.37 | 0.38 | 96.2 | 86.5 |
| 20 | 30 | 0.49 | 0.50 | 96.5 | 87.3 | 0.32 | 0.30 | 0.31 | 95.7 | 87.3 |
| 20 | 40 | 0.49 | 0.50 | 95.3 | 86.3 | 0.22 | 0.20 | 0.20 | 95.1 | 86.3 |
| 30 | 5 | 0.49 | 0.50 | 96.1 | 89.6 | 0.48 | 0.47 | 0.48 | 95.7 | 89.5 |
| 30 | 10 | 0.49 | 0.50 | 96.0 | 89.7 | 0.45 | 0.44 | 0.45 | 96.3 | 89.7 |
| 30 | 20 | 0.49 | 0.50 | 96.1 | 91.0 | 0.40 | 0.38 | 0.39 | 95.9 | 91.0 |
| 30 | 30 | 0.48 | 0.49 | 95.9 | 91.1 | 0.33 | 0.31 | 0.32 | 95.9 | 91.1 |
| 30 | 40 | 0.48 | 0.49 | 94.7 | 89.0 | 0.24 | 0.21 | 0.22 | 93.9 | 89.0 |
| 40 | 5 | 0.48 | 0.49 | 94.9 | 94.4 | 0.48 | 0.46 | 0.47 | 95.6 | 94.4 |
| 40 | 10 | 0.48 | 0.50 | 95.9 | 93.9 | 0.46 | 0.44 | 0.45 | 95.9 | 93.9 |
| 40 | 20 | 0.48 | 0.49 | 95.6 | 92.9 | 0.40 | 0.38 | 0.40 | 95.9 | 92.9 |
| 40 | 30 | 0.47 | 0.49 | 94.7 | 93.4 | 0.34 | 0.31 | 0.33 | 94.6 | 93.4 |
| 40 | 40 | 0.48 | 0.50 | 94.6 | 92.2 | 0.26 | 0.24 | 0.25 | 94.3 | 92.2 |

Data are mean values from 1000 simulated trials for each combination of control arm prevalence of the outcome and non-compliance

**Table S5. Mean absolute bias (log scale) and confidence interval width ratios for simulated trials with at least 80% power to detect an ITT OR=0.5**

|  |  | ITT | | | CACE | | | CI width ratio: CACE to bootstrap ITT | | | CI width ratio: CACE to regression ITT | | | CI width ratio: bootstrap ITT to regression ITT | | |
| --- | --- | --- | --- | --- | --- | --- | --- | --- | --- | --- | --- | --- | --- | --- | --- | --- |
| Control arm prevalence | Non-compliance | Bias | 95% LB | 95% UB | Bias | 95% LB | 95% UB | Ratio | 95% LB | 95% UB | Ratio | 95% LB | 95% UB | Ratio | 95% LB | 95% UB |
| 5 | 5 | -0.029 | -0.045 | -0.014 | -0.032 | -0.049 | -0.015 | 1.075 | 1.072 | 1.078 | 1.442 | 1.435 | 1.449 | 1.341 | 1.335 | 1.347 |
| 5 | 10 | -0.012 | -0.028 | 0.004 | -0.015 | -0.033 | 0.003 | 1.165 | 1.160 | 1.170 | 1.555 | 1.546 | 1.564 | 1.335 | 1.330 | 1.341 |
| 5 | 20 | -0.021 | -0.036 | -0.005 | -0.037 | -0.058 | -0.016 | 1.409 | 1.399 | 1.419 | 1.880 | 1.865 | 1.894 | 1.335 | 1.330 | 1.341 |
| 5 | 30 | -0.027 | -0.041 | -0.012 | -0.078 | -0.104 | -0.052 | 1.855 | 1.835 | 1.875 | 2.472 | 2.445 | 2.500 | 1.336 | 1.330 | 1.342 |
| 5 | 40 | -0.020 | -0.035 | -0.004 | -0.162 | -0.201 | -0.123 | 2.549 | 2.507 | 2.592 | 3.385 | 3.328 | 3.442 | 1.330 | 1.325 | 1.336 |
| 10 | 5 | -0.010 | -0.025 | 0.005 | -0.012 | -0.028 | 0.004 | 1.071 | 1.068 | 1.074 | 1.339 | 1.332 | 1.346 | 1.251 | 1.245 | 1.257 |
| 10 | 10 | -0.026 | -0.041 | -0.011 | -0.031 | -0.048 | -0.013 | 1.153 | 1.148 | 1.157 | 1.448 | 1.439 | 1.456 | 1.257 | 1.250 | 1.263 |
| 10 | 20 | -0.034 | -0.049 | -0.019 | -0.055 | -0.076 | -0.034 | 1.389 | 1.380 | 1.398 | 1.743 | 1.730 | 1.756 | 1.256 | 1.250 | 1.262 |
| 10 | 30 | -0.014 | -0.030 | 0.002 | -0.044 | -0.071 | -0.017 | 1.784 | 1.766 | 1.802 | 2.247 | 2.222 | 2.271 | 1.260 | 1.254 | 1.266 |
| 10 | 40 | -0.022 | -0.037 | -0.007 | -0.146 | -0.183 | -0.110 | 2.575 | 2.541 | 2.609 | 3.227 | 3.183 | 3.271 | 1.257 | 1.251 | 1.263 |
| 20 | 5 | -0.009 | -0.024 | 0.006 | -0.012 | -0.027 | 0.004 | 1.060 | 1.057 | 1.062 | 1.209 | 1.202 | 1.215 | 1.141 | 1.135 | 1.147 |
| 20 | 10 | -0.010 | -0.025 | 0.004 | -0.015 | -0.031 | 0.001 | 1.135 | 1.132 | 1.139 | 1.290 | 1.282 | 1.298 | 1.137 | 1.130 | 1.143 |
| 20 | 20 | -0.011 | -0.026 | 0.003 | -0.022 | -0.041 | -0.002 | 1.336 | 1.329 | 1.342 | 1.520 | 1.510 | 1.531 | 1.139 | 1.133 | 1.145 |
| 20 | 30 | -0.010 | -0.025 | 0.005 | -0.028 | -0.051 | -0.004 | 1.643 | 1.632 | 1.655 | 1.870 | 1.854 | 1.886 | 1.138 | 1.132 | 1.144 |
| 20 | 40 | -0.008 | -0.023 | 0.008 | -0.070 | -0.103 | -0.036 | 2.299 | 2.274 | 2.325 | 2.612 | 2.580 | 2.644 | 1.136 | 1.130 | 1.142 |
| 30 | 5 | -0.005 | -0.020 | 0.010 | -0.004 | -0.020 | 0.011 | 1.047 | 1.045 | 1.050 | 1.122 | 1.116 | 1.129 | 1.072 | 1.067 | 1.078 |
| 30 | 10 | -0.005 | -0.020 | 0.010 | -0.008 | -0.025 | 0.008 | 1.120 | 1.117 | 1.123 | 1.194 | 1.188 | 1.201 | 1.067 | 1.061 | 1.073 |
| 30 | 20 | 0.009 | -0.006 | 0.024 | 0.007 | -0.012 | 0.026 | 1.295 | 1.289 | 1.300 | 1.379 | 1.370 | 1.388 | 1.065 | 1.060 | 1.071 |
| 30 | 30 | -0.009 | -0.024 | 0.006 | -0.029 | -0.051 | -0.007 | 1.562 | 1.553 | 1.571 | 1.660 | 1.648 | 1.673 | 1.064 | 1.058 | 1.069 |
| 30 | 40 | -0.017 | -0.032 | -0.001 | -0.070 | -0.100 | -0.041 | 2.039 | 2.022 | 2.055 | 2.171 | 2.150 | 2.191 | 1.064 | 1.059 | 1.070 |
| 40 | 5 | -0.008 | -0.021 | 0.005 | -0.008 | -0.022 | 0.006 | 1.038 | 1.036 | 1.041 | 1.070 | 1.066 | 1.075 | 1.031 | 1.027 | 1.035 |
| 40 | 10 | -0.015 | -0.029 | 0.000 | -0.016 | -0.032 | 0.000 | 1.100 | 1.096 | 1.103 | 1.123 | 1.117 | 1.129 | 1.022 | 1.017 | 1.026 |
| 40 | 20 | -0.016 | -0.031 | -0.002 | -0.025 | -0.043 | -0.007 | 1.261 | 1.257 | 1.266 | 1.283 | 1.276 | 1.290 | 1.017 | 1.013 | 1.022 |
| 40 | 30 | -0.005 | -0.019 | 0.010 | -0.018 | -0.039 | 0.003 | 1.494 | 1.487 | 1.502 | 1.517 | 1.506 | 1.527 | 1.015 | 1.010 | 1.019 |
| 40 | 40 | -0.009 | -0.024 | 0.006 | -0.050 | -0.077 | -0.023 | 1.855 | 1.844 | 1.866 | 1.877 | 1.863 | 1.891 | 1.012 | 1.007 | 1.016 |

Mean absolute bias = difference between specified parameter OR and median of bootstrapped OR and is shown on the log scale
Data are mean values from 1000 simulated trials for each combination of control arm prevalence of the outcome and non-compliance

**Table S6. Mean absolute bias (log scale) and confidence interval width ratios for simulated trials with at least 90% power to detect an ITT OR=0.5**

|  |  | ITT | | | CACE | | | CI width ratio: bootstrap CACE to bootstrap ITT | | | CI width ratio: bootstrap CACE to regression ITT | | | CI width ratio: bootstrap ITT to regression ITT | | |
| --- | --- | --- | --- | --- | --- | --- | --- | --- | --- | --- | --- | --- | --- | --- | --- | --- |
| Control arm prevalence | Non-compliance | Bias | 95% LB | 95% UB | Bias | 95% LB | 95% UB | Ratio | 95% LB | 95% UB | Ratio | 95% LB | 95% UB | Ratio | 95% LB | 95% UB |
| 5 | 5 | -0.019 | -0.033 | -0.005 | -0.022 | -0.037 | -0.008 | 1.074 | 1.072 | 1.077 | 1.440 | 1.434 | 1.446 | 1.341 | 1.335 | 1.346 |
| 5 | 10 | -0.018 | -0.032 | -0.004 | -0.024 | -0.040 | -0.008 | 1.161 | 1.156 | 1.165 | 1.555 | 1.547 | 1.563 | 1.339 | 1.334 | 1.344 |
| 5 | 20 | -0.012 | -0.026 | 0.002 | -0.021 | -0.040 | -0.002 | 1.391 | 1.383 | 1.399 | 1.865 | 1.853 | 1.877 | 1.341 | 1.336 | 1.347 |
| 5 | 30 | -0.014 | -0.028 | 0.000 | -0.046 | -0.070 | -0.023 | 1.805 | 1.787 | 1.823 | 2.423 | 2.398 | 2.448 | 1.343 | 1.338 | 1.348 |
| 5 | 40 | -0.023 | -0.038 | -0.009 | -0.145 | -0.181 | -0.109 | 2.622 | 2.586 | 2.657 | 3.510 | 3.461 | 3.559 | 1.341 | 1.336 | 1.347 |
| 10 | 5 | -0.001 | -0.015 | 0.012 | -0.002 | -0.016 | 0.012 | 1.068 | 1.066 | 1.070 | 1.360 | 1.353 | 1.367 | 1.274 | 1.268 | 1.280 |
| 10 | 10 | -0.012 | -0.025 | 0.001 | -0.016 | -0.031 | -0.001 | 1.149 | 1.145 | 1.153 | 1.471 | 1.463 | 1.478 | 1.280 | 1.275 | 1.286 |
| 10 | 20 | -0.019 | -0.032 | -0.006 | -0.035 | -0.053 | -0.017 | 1.366 | 1.359 | 1.373 | 1.740 | 1.729 | 1.751 | 1.274 | 1.269 | 1.280 |
| 10 | 30 | -0.012 | -0.025 | 0.002 | -0.039 | -0.062 | -0.017 | 1.721 | 1.708 | 1.733 | 2.185 | 2.167 | 2.203 | 1.270 | 1.264 | 1.276 |
| 10 | 40 | -0.029 | -0.043 | -0.016 | -0.150 | -0.183 | -0.117 | 2.545 | 2.513 | 2.577 | 3.230 | 3.187 | 3.273 | 1.271 | 1.266 | 1.277 |
| 20 | 5 | -0.003 | -0.016 | 0.010 | -0.007 | -0.021 | 0.007 | 1.058 | 1.056 | 1.060 | 1.234 | 1.227 | 1.241 | 1.167 | 1.161 | 1.173 |
| 20 | 10 | -0.010 | -0.024 | 0.003 | -0.015 | -0.030 | -0.000 | 1.132 | 1.129 | 1.135 | 1.315 | 1.308 | 1.322 | 1.162 | 1.157 | 1.168 |
| 20 | 20 | -0.011 | -0.024 | 0.003 | -0.022 | -0.039 | -0.004 | 1.327 | 1.322 | 1.333 | 1.541 | 1.531 | 1.551 | 1.161 | 1.155 | 1.168 |
| 20 | 30 | -0.005 | -0.018 | 0.009 | -0.027 | -0.049 | -0.006 | 1.626 | 1.616 | 1.635 | 1.876 | 1.862 | 1.890 | 1.154 | 1.148 | 1.160 |
| 20 | 40 | -0.009 | -0.022 | 0.005 | -0.068 | -0.097 | -0.039 | 2.240 | 2.219 | 2.261 | 2.596 | 2.568 | 2.624 | 1.159 | 1.153 | 1.165 |
| 30 | 5 | -0.002 | -0.015 | 0.011 | -0.005 | -0.018 | 0.008 | 1.049 | 1.046 | 1.051 | 1.137 | 1.131 | 1.142 | 1.085 | 1.080 | 1.090 |
| 30 | 10 | -0.004 | -0.016 | 0.009 | -0.007 | -0.021 | 0.008 | 1.113 | 1.110 | 1.116 | 1.206 | 1.200 | 1.212 | 1.084 | 1.079 | 1.089 |
| 30 | 20 | -0.006 | -0.019 | 0.006 | -0.013 | -0.028 | 0.003 | 1.283 | 1.279 | 1.288 | 1.393 | 1.385 | 1.400 | 1.085 | 1.080 | 1.090 |
| 30 | 30 | -0.021 | -0.035 | -0.008 | -0.041 | -0.060 | -0.021 | 1.532 | 1.524 | 1.539 | 1.651 | 1.640 | 1.661 | 1.078 | 1.073 | 1.083 |
| 30 | 40 | -0.022 | -0.036 | -0.008 | -0.076 | -0.103 | -0.049 | 1.987 | 1.974 | 2.000 | 2.146 | 2.129 | 2.163 | 1.080 | 1.075 | 1.085 |
| 40 | 5 | -0.011 | -0.023 | 0.001 | -0.013 | -0.025 | -0.001 | 1.039 | 1.037 | 1.041 | 1.081 | 1.077 | 1.085 | 1.041 | 1.037 | 1.045 |
| 40 | 10 | -0.008 | -0.020 | 0.004 | -0.013 | -0.026 | 0.000 | 1.099 | 1.097 | 1.102 | 1.135 | 1.130 | 1.139 | 1.033 | 1.029 | 1.036 |
| 40 | 20 | -0.012 | -0.024 | 0.001 | -0.021 | -0.036 | -0.006 | 1.257 | 1.253 | 1.260 | 1.294 | 1.288 | 1.300 | 1.030 | 1.026 | 1.034 |
| 40 | 30 | -0.022 | -0.034 | -0.009 | -0.043 | -0.061 | -0.025 | 1.475 | 1.469 | 1.481 | 1.519 | 1.511 | 1.527 | 1.030 | 1.026 | 1.034 |
| 40 | 40 | -0.010 | -0.023 | 0.003 | -0.046 | -0.068 | -0.023 | 1.825 | 1.815 | 1.834 | 1.872 | 1.860 | 1.883 | 1.026 | 1.022 | 1.029 |

Mean absolute bias = difference between specified parameter OR and median of bootstrapped OR and is shown on the log scale
Data are mean values from 1000 simulated trials for each combination of control arm prevalence of the outcome and non-compliance

**Supplementary methods**

1. ***Data generation mechanism***

The number of clusters required for each scenario was calculated using an initial set of simulations following the same data generating mechanism described in the main paper.

Cluster-level prevalence was achieved by sampling from the distribution

$$\left[ \begin{matrix} a' \\ d' \end{matrix} \right]\sim N\left( \left[ \begin{matrix} 0 \\ 0 \end{matrix} \right],\left[ \begin{matrix} 1 & 0.5 \\ 0.5 & 1 \end{matrix} \right] \right)$$

These values were then transformed to the beta distribution. The shape parameters were determined as:

$$\alpha=\frac{p\left[ p\left( 1-p \right)-\sigma_{b}^{2} \right]}{\sigma_{b}^{2}}$$

$$\beta=\frac{\left[ 1-p \right]\left[ p\left( 1-p \right)-\sigma_{b}^{2} \right]}{\sigma_{b}^{2}}$$

Where $p$ is the prevalence of non-adherence, baseline prevalence, or prevalence after exposure to intervention, and $\sigma_{b}^{2}$ is the value of between cluster variability to give each prevalence a coefficient of variation (defined as $\sigma_{b}/p$) of 0.2.

We are assuming that the true cluster proportions $p_{i}$ follow a beta distribution with mean $p$ and variance $\sigma_{b}^{2}.$ The beta distribution is specified in terms of shape parameters $\alpha$ and $\beta$ <https://en.wikipedia.org/wiki/Beta_distribution>.

Also: DeGroot, M. H., & Schervish, M. J. (2012). *Probability and statistics* (4th ed.). Pearson Education.

$$E\left[ p_{i} \right]=p=\frac{\alpha}{\alpha+\beta}$$

And

$$Var\left[ p_{i} \right]=\sigma_{b}^{2}=\frac{\alpha\beta}{\left( \alpha+\beta\right)^{2}\left( \alpha+\beta+1 \right)}$$

From these simultaneous equations, rearrange so that:

$$\beta=\frac{\alpha\left( 1-p \right)}{p}$$

Then substitution:

$$\sigma_{b}^{2}=\frac{\alpha\frac{\alpha\left( 1-p \right)}{p}}{\left( \alpha+\frac{\alpha\left( 1-p \right)}{p} \right)^{2}\left( \alpha+\frac{\alpha\left( 1-p \right)}{p}+1 \right)}$$

This gives

$$\alpha=\frac{p\left[ p\left( 1-p \right)-\sigma_{b}^{2} \right]}{\sigma_{b}^{2}}$$

Then substituting this into the formula for $\beta$:

$$\beta=\frac{\left[ 1-p \right]\left[ p\left( 1-p \right)-\sigma_{b}^{2} \right]}{\sigma_{b}^{2}}$$

For fixed proportion of non-adherence ($a$), proportion of disease at baseline ($d_{n}$), and ITT odds ratio ($OR_{ITT}$), the CACE odds ratio for each scenario was determined using the following formula:

$$OR{}_{CACE}=Q_{IA}\left[ \frac{\left( OR_{ITT}+Q_{I} \right)\left( a+\left( 1-a \right)d_{n} \right)-Q_{I}}{Q_{I}-\left( 1-a \right)d_{n}\left( OR_{ITT}+Q_{I} \right)} \right]$$

Where $Q_{IA}$ is the odds of disease in adherent individuals in the intervention arm calculated as:

$$Q_{IA}=\frac{d_{IA}}{1-d_{IA}}$$

$$d_{IA}=\frac{d_{n}\left( OR_{ITT}-\left[ 1-a \right]\left[ 1-d_{n}\left\{ 1-OR_{ITT} \right\} \right] \right)}{a\left( 1-d_{n}[1-OR_{ITT}] \right)}$$

And $Q_{I}$ is the odds of disease in the intervention arm calculated as:

$$Q_{I}=\frac{d_{I}}{1-d_{I}}$$

$$d_{I}=\left( 1-a \right)d_{n}+ad_{IA}$$

This assumes that disease prevalence at baseline is independent of non-adherence. Whilst this is not true in our simulation study, the value of OR_ITT_ was only used to estimate the number of clusters needed to achieve 80% power. The derived OR_CACE_ was used directly in the simulation study.

1. **Stata code and data**

**Data format required: in the example code below, it is assumed the data are saved as a Stata dataset call “crt_data”, structured as follows;**

| Cluster ID (cl_id | Arm (arm; 1 = intervention, 0 = no intervention) | Individual unique ID (id) | Non-compliance (np: 0=complier, 1 = non-complier, observed in intervention arm) | Outcome at follow-up (out2; 0 = negative, 1= positive) |
| --- | --- | --- | --- | --- |
| 1 | Intervention | 1 | 1 | 0 |
| 1 | Intervention | 1 | 1 | 0 |
| 1 | Intervention | 2 | 0 | 0 |
| 1 | Intervention | 3 | 0 | 0 |
| 1 | Intervention | 4 | 0 | 0 |
| 1 | Intervention | 5 | 0 | 1 |
| 1 | Intervention | 6 | 0 | 0 |
| … |  |  |  |  |
| 4 | Intervention | 301 | 0 | 0 |
| 4 | Intervention | 302 | 0 | 1 |
| 4 | Intervention | 303 | 1 | 0 |
| 4 | Intervention | 304 | 1 | 1 |
| 4 | Intervention | 305 | 0 | 1 |
| 4 | Intervention | 306 | 0 | 0 |
| …. |  |  |  |  |
| 11 | No intervention | 1001 |  | 0 |
| 11 | No intervention | 1002 |  | 0 |
| 11 | No intervention | 1003 |  | 0 |
| 11 | No intervention | 1004 |  | 1 |
| 11 | No intervention | 1005 |  | 1 |
| 11 | No intervention | 1006 |  | 0 |
| …. |  |  |  |  |

Note: the unique ID for individuals can take any format, this is not required in the code below, just that the data need to include individual observations

**Stata code:**

********************************************************************

* STEP 1: 1,000 BOOTSTRAP SAMPLING TO GET CACE SAMPLING DISTRIBUTION

* STEP 2: EXTRACT BOOTSTRAP RESULTS TO DISPLAY CACE (95% CI)

********************************************************************

* CHANGE DIRECTORY TO WORKING FOLDER

cd "..."

* OPEN THE DATASET: INDIVIDUAL LEVEL CRT DATA

use "crt_data", clear

********************************************************************

* STEP 1: 1,000 BOOTSTRAP SAMPLES TO GET CACE SAMPLING DISTRIBUTION

********************************************************************

* PROGRAM TO RUN BOOTSTRAP SAMPLING:

capture program drop crt_cace_boot

program define crt_cace_boot, rclass

cap gen count=1

preserve

bsample, strata(arm cl_id np)

* collapse the data:

sort cl_id arm np

by cl_id arm np: gen sumout2 = sum(out2)

drop out2

rename sumout2 out2

by cl_id arm np: gen n = sum(count)

by cl_id arm np: keep if _n == _N

tab arm np,m

gen out_prev=out2/n

summ

summ out_prev, detail

bysort arm: summ out2, detail

bysort arm: summ out_prev, detail

replace np=. if arm==0

summ

* summarise intervention arm arm:

tabstat out2 n if arm==1, stats(sum) by(np) save

// np: 0=complier, 1=non-complier

matrix m_int_c=r(Stat1)

matrix list m_int_c

local r_int_c=m_int_c[1,1]

local n_int_c=m_int_c[1,2]

* prev in Cs in INTERVENTION arm

local p_int_c=`r_int_c'/`n_int_c'

matrix m_int_nc=r(Stat2)

matrix list m_int_nc

local r_int_nc=m_int_nc[1,1]

local n_int_nc=m_int_nc[1,2]

* prev in NCs in INTERVENTION arm

local p_int_nc=`r_int_nc'/`n_int_nc'

matrix m_int_t=r(StatTotal)

matrix list m_int_t

local r_int_t=m_int_t[1,1]

local n_int_t=m_int_t[1,2]

* overall rate in the INTERVENTION arm

local p_int_t=`r_int_t'/`n_int_t'

* summarise control arm: (overall)

tabstat out2 n if arm==0, stats(sum) save

matrix m_con_t=r(StatTotal)

matrix list m_con_t

local r_con_t=m_con_t[1,1]

local n_con_t=m_con_t[1,2]

* overall risk in the control arm

local p_con_t=`r_con_t'/`n_con_t'

* #(NPs in control arm) =

local n_con_nc=(`n_int_nc'/`n_int_t')*`n_con_t'

local n_con_c=`n_con_t'-`n_con_nc'

local p_con_nc=`p_int_nc'

local r_con_nc=`n_con_nc'*`p_con_nc'

local r_con_c=`r_con_t'-`r_con_nc'

* risk in control arm compliers:

local p_con_c=`r_con_c'/`n_con_c'

* ITT ODDS RATIO:

local orb_itt=(`p_int_t'/(1-`p_int_t'))/(`p_con_t'/(1-`p_con_t'))

return scalar orb_itt=`orb_itt'

* CACE RATE RATIO:

local orb_cace=(`p_int_c'/(1-`p_int_c'))/(`p_con_c'/(1-`p_con_c'))

return scalar orb_cace=`orb_cace'

disp `orb_itt'

disp `orb_cace'

restore

end

* RUN PROGRAM ON CRT DATASET:

bootstrap r(orb_itt) r(orb_cace), cluster(cl_id) strata(arm) reps(1000) nowarn saving("crt_data_boot", every(1) replace): crt_cace_boot

*************************************************************

* STEP 2: EXTRACT BOOTSTRAP RESULTS TO DISPLAY CACE (95% CI)

*************************************************************

use "crt_data_boot", clear

rename _bs_1 orb_itt

rename _bs_2 orb_cace

* BOOTSTRAPPED CACE (95% CI):

_pctile orb_cace, p(2.5, 50, 97.5)

local oc25=r(r1)

local oc50=r(r2)

local oc975=r(r3)

disp "CACE (95% CI): " `oc50' " (" `oc25' ", " `oc975' ")"

hist orb_cace, graphregion(color(white)) normal

* BOOTSTRAPPED ITT OR (95% CI):

* FOR CHECKING PURPOSES; IS IT SIMILAR TO ITT ANALYSIS

_pctile orb_itt, p(2.5, 50, 97.5)

local oi25=r(r1)

local oi50=r(r2)

local oi975=r(r3)

disp "BOOTSTRAPPED ITT OR (95% CI): " `oi50' " (" `oi25' ", " `oi975' ")"

* check the distribution of the sampling distribution for the CACE bootstrapped values

hist orb_itt, graphregion(color(white)) normal
